# Dynamics of IL-4 and IL-13 expression in the airways of sheep following allergen challenge

**DOI:** 10.1186/s12890-015-0097-9

**Published:** 2015-09-11

**Authors:** Bahar Liravi, David Piedrafita, Gary Nguyen, Robert J. Bischof

**Affiliations:** Biotechnology Research Laboratories, Department of Physiology, Monash University, Clayton, 3800 VIC Australia; School of Applied and Biomedical Sciences, Federation University, Churchill, 3842 VIC Australia; The Ritchie Centre, Hudson Institute of Medical Research, Clayton, 3168 VIC Australia

## Abstract

**Background:**

IL-4 and IL-13 play a critical yet poorly understood role in orchestrating the recruitment and activation of effector cells of the asthmatic response and driving the pathophysiology of allergic asthma. The house dust mite (HDM) sheep asthma model displays many features of the human condition and is an ideal model to further elucidate the involvement of these critical Th_2_ cytokines. We hypothesized that airway exposure to HDM allergen would induce or elevate the expression profile of IL-4 and IL-13 during the allergic airway response in this large animal model of asthma.

**Methods:**

Bronchoalveolar lavage (BAL) samples were collected from saline- and house dust mite (HDM)- challenged lung lobes of sensitized sheep from 0 to 48 h post-challenge. BAL cytokines (IL-4, IL-13, IL-6, IL-10, TNF-α) were each measured by ELISA. IL-4 and IL-13 expression was assessed in BAL leukocytes by flow cytometry and in airway tissue sections by immunohistology.

**Results:**

IL-4 and IL-13 were increased in BAL samples following airway allergen challenge. HDM challenge resulted in a significant increase in BAL IL-4 levels at 4 h compared to saline-challenged airways, while BAL IL-13 levels were elevated at all time-points after allergen challenge. IL-6 levels were maintained following HDM challenge but declined after saline challenge, while HDM administration resulted in an acute elevation in IL-10 at 4 h but no change in TNF-α levels over time. Lymphocytes were the main early source of IL-4, with IL-4 release by alveolar macrophages (AMs) prominent from 24 h post-allergen challenge. IL-13 producing AMs were increased at 4 and 24 h following HDM compared to saline challenge, and tissue staining provided evidence of IL-13 expression in airway epithelium as well as immune cells in airway tissue.

**Conclusion:**

In a sheep model of allergic asthma, airway inflammation is accompanied by the temporal release of key cytokines following allergen exposure that primarily reflects the Th_2_-driven nature of the immune response in asthma. The present study demonstrates for the first time the involvement of IL-4 and IL-13 in a relevant large animal model of allergic airways disease.

## Background

Asthma is a chronic inflammatory disease of the lungs characterized by inflammation, airway hyperresponsiveness (AHR) and airway wall remodelling. Atopic asthmatics display high levels of allergen-specific immunoglobulin E (IgE) antibodies, and this is associated with the development of a type 2 immune response with evidence of elevated expression levels of T-helper type 2 (Th_2_) cytokines [1].

The Th_2_ cytokines, including interleukin (IL)-4, IL-5, IL-9, IL-13 and IL-25, together promote key pathophysiological features of asthma including allergen-specific IgE, airway inflammation (characterized by activated lymphocytes, eosinophils, mast cells and macrophages), damage to the airway epithelium, mucus gland hyperplasia and structural remodelling of the airway wall [2–4]. Other cytokines have been implicated in the pathogenesis of asthma. For example, IL-6 is a marker of inflammation and serves as an important regulator of effector CD4^+^ T cell fate by promoting IL-4 production during Th_2_ differentiation while inhibiting Th1 differentiation [5]. An immunoregulatory role for IL-6 in asthma and other pulmonary diseases where the lung epithelium is damaged has been highlighted [6].

There is strong evidence that IL-4 and IL-13 play a crucial role in orchestrating the recruitment and activation of the effector cells of the asthmatic response. IL-4 is an essential trigger for Th_2_ lymphocyte differentiation, and both IL-4 and IL-13 can induce IgE class switching in B lymphocytes. Additionally, IL-4 and IL-13 act on bronchial epithelial, endothelial and airway smooth muscle cells, collectively leading to many of the pathophysiological features of asthma [3, 7].

Clinical observations of IL-4 in allergic asthma include increased IL-4 in the serum and bronchoalveolar lavage (BAL) of allergic individuals [8, 9], while nebulized IL-4 given to patients with mild asthma results in a significant increase in AHR associated with the elevation of sputum eosinophil numbers [10]. Studies using IL-4 deficient mice indicate that the main role of IL-4 in allergic airway inflammation is during the initial priming of Th_2_ effector cells [3, 11].

In murine and human studies, IL-13 has been shown to be directly responsible for eosinophil survival and proliferation within lung tissue, the release of mediators responsible for bronchoconstriction and the induction of mucus hypersecretion (reviewed in [12]). IL-13 mRNA expression has been reported in bronchial biopsies from both allergic and non-allergic asthmatic subjects [13], and IL-13 in BAL following allergen provocation of asthmatic subjects is strongly correlated with an increase in eosinophil numbers [14, 15]. In mice, human IL-13 promotes many of the inflammatory changes associated with asthma, such as inflammatory cell infiltration in the lungs and AHR and goblet cell metaplasia [16, 17]; treatment with an anti- IL-13 monoclonal antibody (mAb) has been shown to be effective in mice [18], although poorer outcomes have been realised to date in human clinical trials [19, 20].

A sheep model of asthma based on the relevant allergen house dust mite (HDM) displays many key features of the human asthmatic condition [21, 22]. Sheep sensitized to HDM develop allergen-specific IgE responses, inflammation of the airways following airway allergen challenge that includes profound eosinophilia, airway epithelial mucus hypersecretion, airway wall remodelling and early and late-phase asthmatic responses and AHR following airway allergen challenge [22–24]. Unfortunately, our current understanding of the role of Th_2_cytokines in larger animal models of asthma, where experimental therapeutic and in real-time manipulation is viable, is limited to a description or phenotypic analysis of inflammatory cells involved in the allergic response (reviewed in [25, 26]), and a single study in non-human primates reporting elevated mRNA expression of IL-4 and IL-13 in HDM-challenged airways [27].

The current study aimed to investigate the expression of IL-4 and IL-13, as well as IL-6, IL-10 and TNF-α, in the sheep model of asthma following allergen challenge of the airways. We hypothesized that airway allergen challenge would induce an elevated expression profile of two critical Th_2_ cytokines, IL-4 and IL-13, in the HDM sheep model of allergic asthma.

## Methods

### Animals and HDM sensitization and challenge protocols

Merino-cross ewes (8–12 months old), treated orally with anthelminthic to eliminate any worm parasites prior to the experiment, were housed in indoor pens and fed *ad libitum*. All experimental work was approved by the Monash University Animal Ethics Committee.

**Fig. 1:**
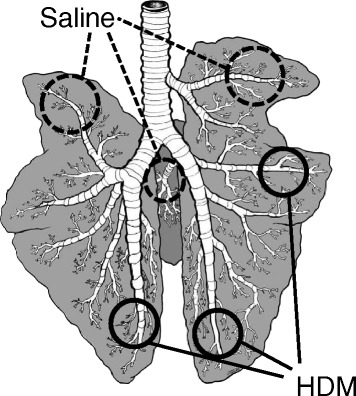
Ovine lung diagram indicating the location of lung segments used for the bronchoscopic delivery of HDM (solid line) and saline control (broken line) treatments within the same sheep

Sheep were sensitized by subcutaneous immunizations with a solubilized preparation of house dust mite extract (HDM; CSL Ltd, Parkville, Australia) as detailed elsewhere [23, 28]. Sensitized animals (HDM-specific IgE responders) were rested for 3 weeks, then given segmental airway challenges with HDM (500 μg in 5 ml) at weekly intervals over 3 weeks (ie. three challenges over 3 weeks) using a flexible fibre-optic endoscope (Model FG-16×, Pentax, NJ, USA). The airway challenges for each animal involved a segmental bolus infusion of 5 ml HDM solution into each of 3 discrete lung lobes (HDM administered only to the left caudal, right caudal and right middle lobes; see Fig. 1 and [22]) and a 5 ml infusion of sterile saline into each of three control lung lobes (saline administered only to the left cranial, right cranial and accessory lobes; Fig. 1 and [22]). Thus, over the entire period of airway challenges, each separate lung lobe was exposed to only saline or HDM; further, in each animal and on each challenge occasion, HDM and saline segmental challenges were given at the same time-point. At the time of the third and final airway challenge with saline/HDM, BAL collections were performed on individual lobes for subsequent cellular and cytokine analyses (as detailed below).

### Bronchoalveolar lavage (BAL) sampling

On the occasion of the final (3rd) airway challenge, BAL collections with sterile saline were performed on separate lung lobes at 0, 4, 24 and 48 h following airway saline/HDM challenge using a fibre-optic endoscope [23, 28] and samples placed on ice before being centrifuged at 400 *g* to separate BAL cells from BAL fluid. BAL fluid samples were concentrated by centrifugation filtration using 3 kDa nominal molecular weight limit devices (Amicon Ultra-15, Millipore, Bedford, MA, USA) following the manufacturer instructions and stored at −80 °C prior to cytokine quantitation (see below). Pelleted BAL cells were resuspended in 5 ml of sterile PBS and used for flow cytometry (see below). Total cell counts were determined using a haemocytometer and presented as cells/ml BAL fluid. Differential cell counts (total of 200 cells) were performed on cytospot preparations stained with Kwik Diff^TM^ solution (Thermo Fisher Scientific, MA, USA) to enumerate proportions of macrophages, lymphocytes, eosinophils and neutrophils.

### Cytokine detection in BAL samples

Protein levels of IL-4, IL-6, IL-10, IL-13 and TNF-α in BAL samples were determined using ovine-specific ELISAs. Antibodies and standards used are detailed in Table 1, with IL-4, IL-6, IL-10 and TNF-α ELISA protocols as previously reported [29–31], and the IL-13 ELISA protocol developed and optimized in-house.

**Table 1:** Details of reagents used in ovine-specific cytokine ELISAs

ELISA	Antibody (Ab)/cytokine details	Clone/type	Dilution	Source
IL-4	*rov* IL-4 (cytokine standard)	-	-	Moredun^a^
	Anti-bovine IL-4 mAb (coating)	CC313	1:400	AbD Serotec^b^
	Anti-bovine IL-4/biotinylated mAb (detecting)	CC314	1:1600	AbD Serotec
	Streptavidin/HRP	-	1:1000	Dako^c^
IL-6	*rov* IL-6 (cytokine standard)	-	-	CAB^d^
	Anti-ovine IL-6 mAb (coating)	4B6	1:400	Epitope^e^
	Anti-ovine IL-6, pAb (detecting)	rabbit pAb	1:5000	CAB
	Anti-rabbit Ig/HRP	-	1:1000	Dako
IL-10	*rbov* IL-10 (cytokine standard)	-	-	Moredun
	Anti-bovine IL-10 mAb (coating)	CC318	1:200	AbD Serotec
	Anti-bovine IL-10/biotinylated mAb (detecting)	CC320	1:1000	AbD Serotec
	Streptavidin-HRP	-	1:1000	Dako
IL-13	*rbov* IL-13 (cytokine standard)	-	-	Kingfisher^f^
	Anti-bovine IL-13, pAb (coating)	rabbit pAb	1:800	Kingfisher
	Anti-bovine IL-13/biotinylated pAb (detecting)	rabbit pAb	1:2000	Kingfisher
	Streptavidin/HRP	-	1:1000	Dako
TNFα	*rov* TNFα (cytokine standard)	-	-	CAB
	Anti-ovine TNFα mAb ascites (coating)	6.09	1:200	CAB
	Anti-ovine TNFα pAb (detecting)	rabbit pAb	1:500	CAB
	Anti-rabbit Ig/HRP	-	1:1000	Dako

*rov* Recombinant ovine, *rbov* Recombinant bovine, *mAb* Monoclonal antibody, *pAb* polyclonal antibody

^a^Moredun: Dr Gary Entrican, Moredun Research Institute, Edinburgh, Scotland

^b^AbD Serotec: AbD Serotec, UK

^c^Dako: Agilent Technologies Inc., CA, USA

^d^CAB: Centre for Animal Biotechnology, School of Veterinary Science, Melbourne, Australia

^e^Epitope: Epitope Technologies Pty Ltd, VIC, Australia

^f^Kingfisher: Kingfisher Biotech, Inc, MN, USA

### BAL cell characterization by flow cytometry

For intracellular cytokine staining, BAL cells were resuspended in cell culture medium (DMEM containing 10 % fetal bovine serum (FBS) and 0.1 % gentamycin; Life Technologies, CA, USA) at 10^6^ cells/ml and incubated in the presence of Brefeldin A (20 μg/ml; Cell Signaling Technology, MA, USA) for 2 h, then washed and resuspended in intracellular blocking buffer (PBS, 0.1 % saponin, 5 % normal horse serum, 5 % normal sheep serum (NSS), 5 % FBS, 0.01 % sodium azide) to reduce non-specific binding. Cells were then incubated for 30 min at 4 °C with antibodies (Abs) to IL-4 (FITC-conjugated anti-bovine IL-4 mAb, 1:100; AbD Serotec, UK), or IL-13 (rabbit anti-bovine IL-13 polyclonal antibody (pAb), 1:100; Kingfisher Biotech Inc., MN, USA) with matching secondary Ab (Alexa Fluor^TM^ 488 goat anti-rabbit IgG, 1:200; Jackson ImmunoResearch Laboratories Inc., PA, USA). Cells were washed and fixed in 2 % paraformaldehyde (PFA; Fluka, St. Gallen, Switzerland) in PBS and stored at 4 °C prior to analysis by flow cytometry. Leukocyte cell populations were gated on the basis of forward scatter (FSC) and side scatter (SSC) characteristics [23] and 10,000 events were acquired using a BD LSR II flow cytometer (Becton Dickinson Biosciences, CA, USA), with data analyzed using FlowJo^TM^ software (TreeStar Inc, OR, USA).

### Immunostaining of lung tissue

Lung tissues were collected at post-mortem, 48 h post-allergen challenge, and embedded in Optimal Cutting Temperature (OCT) medium (Tissue Tek, Miles Inc., PA, USA) and stored at −80 °C prior to immunostaining. Frozen tissue sections (5 μM) were cut onto glass microscope slides, then air-dried and fixed in 2 % PFA. Slides were washed in PBS/ 0.1 % Tween 20 and incubated with anti- IL-4 (anti-bovine IL-4 mAb, 1:100, clone CC313; AbD Serotec) or a combination of anti- IL-13 (as detailed above) and anti-CD45 (common leukocyte marker; [32]) antibodies. Secondary Abs included Alexa Fluor^TM^ 594 anti-mouse Ig (1:1000; Life Technologies) for IL-4 and CD45, and Alexa Fluor^TM^ 488 anti-rabbit IgG (1:200; Jackson ImmunoResearch) for IL-13 detection. Slides were then washed and mounted with Mowiol mounting medium (Calbiochem, CA, USA) containing 4′, 6-diamidino-2-phenylindole, dihydrochloride (DAPI, 1:5000; Life Technologies).

#### Statistical analysis

To assess the effect of saline or HDM challenge as well as time following challenge on BAL cell numbers, cell percentages, cytokine levels and cytokine expressing cells, a two-way ANOVA test was performed followed by a Holm-Sidak test to correct for multiple comparisons. Differences were considered significant for *p* < 0.05. All data are reported as the mean ± standard deviation (SD).

## Results

### Analysis of BAL cells following airway allergen challenge

**Fig. 2:**
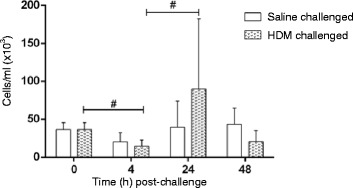
Kinetics of leukocyte traffic into BAL fluid over time following saline and HDM bronchial challenges. Data presented as mean counts (cells/ml BAL) ± SD for *n* = 6 sheep (^#^denotes significant difference; ^#^ *p* < 0.05)

BAL leukocyte numbers from HDM- and saline- challenged lungs before and at 4, 24 and 48 h post-challenge are shown in Fig. 2. In saline-challenged lobes there was little change in leukocyte numbers across the different time points. Conversely, in HDM-challenged lung lobes there was initially a decline in total BAL leukocyte number at 4 h, followed by a significant increase by 24 h post-challenge (Fig. 2).

**Fig. 3:**
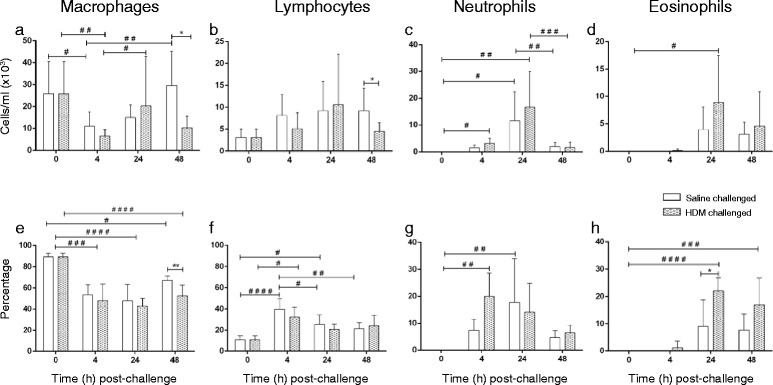
Kinetics of leukocyte sub-population traffic into BAL fluid over time following saline and HDM bronchial challenges, showing changes in (**a**–**d**) cell number and (**e**–**h**) cell percentage over time. Data presented as mean counts ± SD for *n* = 6 sheep (*denotes significant difference between HDM and saline-challenged lobes at the corresponding time point: *p < 0.05, **p < 0.01; ^#^denotes significant difference between time points: ^#^ *p* < 0.05, ^##^ *p* < 0.01, ^###^ *p* < 0.001, ^####^ *p* < 0.0001)

While macrophages were the major cell component in BAL, there was an initial decline in numbers at 4 h after saline or HDM challenge, followed by a return to similar baseline levels within 48 h post-challenge (Fig. 3a). Macrophages represented ~90 % of BAL at baseline but this was reduced at all time points post-challenge (Fig. 3e). Lymphocyte numbers showed little change over time (Fig. 3b), although the percentage of lymphocytes in BAL increased at 4 h in saline- and HDM- challenged lobes compared to pre-challenge (Fig. 3f). There was a significant increase in neutrophils in BAL over the first 4–24 h after saline or HDM challenge (Fig. 3c, g). In contrast, eosinophils that were absent in BAL prior to airway challenge were recruited into BAL following HDM challenge, reaching their maximum at 24–48 h (Fig. 3d, h).

### BAL cytokine profiles

**Fig. 4:**
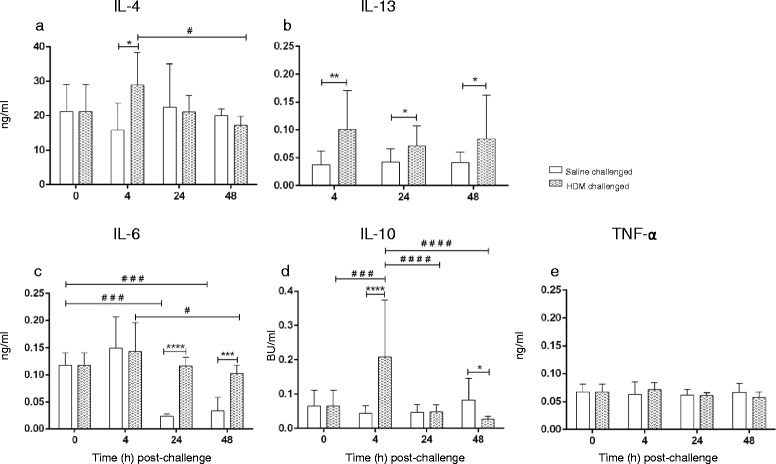
Cytokine protein levels over time (0–48 h) showing (**a**) IL-4, (**b**) IL-13, (**c**) IL-6, (**d**) IL-10 and (E) TNF-α in BAL fluid following saline and HDM bronchial challenges. Data presented as means ± SD for *n* = 10 sheep (*denotes significant difference between HDM and saline-challenged lobes at the corresponding time point: *p < 0.05, **p < 0.01, ***p < 0.001, ****p < 0.0001; ^#^denotes significant difference between time points: ^#^ *p* < 0.05, ^##^ *p* < 0.01, ^###^ *p* < 0.001, ^####^ *p* < 0.0001)

IL-4, IL-13, IL-6, IL-10 and TNF-α levels were assessed in BAL samples collected after saline or HDM challenge (Fig. 4). BAL IL-4 levels were greater in HDM-challenged lobes compared to saline at 4 h but decreased between 4 and 48 h after HDM challenge (Fig. 4a). IL-13 levels in HDM-challenged lobes were significantly higher than saline-challenged lobes at each of the time points examined (Fig. 4b). IL-6 levels at 4 h post-challenge showed no significant difference between HDM and saline treated lobes, while greater levels of IL-6 were detected in HDM-challenged lobes compared to saline at 24 and 48 h (Fig. 4c). Further, there was a significant decline in BAL IL-6 levels from the 0 h/4 h time-point to 24 and 48 h after saline challenge. IL-10 levels were increased at 4 h and decreased at 48 h in HDM-compared to saline-challenged lobes (Fig. 4d). TNF-α levels in HDM-challenged lobes were similar to saline-challenged lobes at the examined time points and there was no change in BAL over time (Fig. 4e).

### Intracellular expression of IL-4 and IL-13 in BAL cells

BAL cell subpopulations were identified in the pre- (0 h) and post-challenge (4 h, 24 h, 48 h) BAL samples, with gating based on forward- and side- light scatter (FSC, SSC) properties as reported elsewhere [23]. BAL cell subpopulations were then examined for intracellular IL-4 and IL-13 expression and analyzed by flow cytometry.

**Fig. 5:**
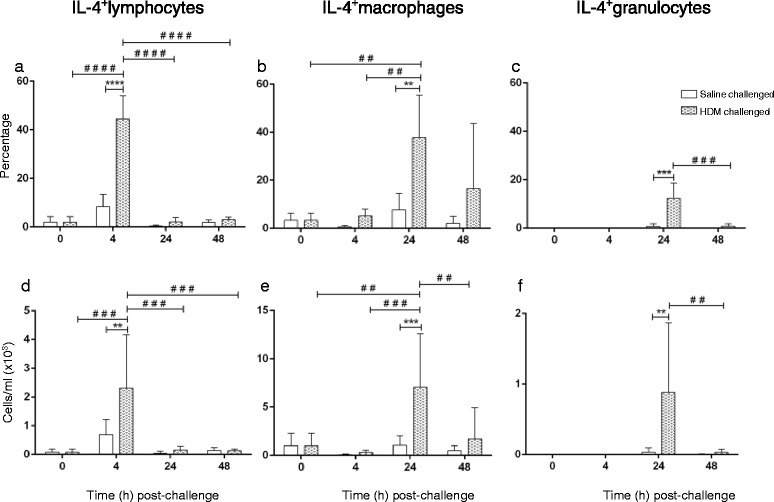
IL-4 detection in BAL cells following saline and HDM bronchial challenges. **a**–**c** percentage and (**d**–**f**) total numbers (cells/ml BAL) of IL-4 expressing lymphocytes, macrophages and granulocytes collected at 0 h (baseline), and at 4, 24 and 48 h post-segmental airway challenge. Data presented as mean positive cells (within subpopulation) ± SD for *n* = 6 sheep (*denotes significant differences; *p < 0.05, **p < 0.01, ***p < 0.001, ****p < 0.0001; ^#^denotes significant difference between time points: ^##^ *p* < 0.01, ^###^ *p* < 0.001, ^####^ *p* < 0.0001)

There was a marked but transient increase in the proportion and total number of IL-4^+^ lymphocytes from 0 to 4 h in HDM-challenged lungs, followed by a return to baseline levels from 24 h post-challenge; the increase in IL-4 expression at 4 h was significant compared to saline-challenged lobes at that time-point (Fig. 5a & d). There was a significant increase in the percentage and total number of IL-4^+^ macrophages at 24 h post-HDM challenge compared to earlier time-points and saline challenge (at 24 h), with a subsequent decline to baseline levels at 48 h post-challenge (Fig. 5b & e). Of the granulocytes recruited into the lungs at 24 h post-HDM challenge, the proportion and the total number of these cells expressing IL-4 was greater in HDM- compared to saline-challenged lobes, although no difference was observed at 48 h (Fig. 5c & f).

**Fig. 6:**
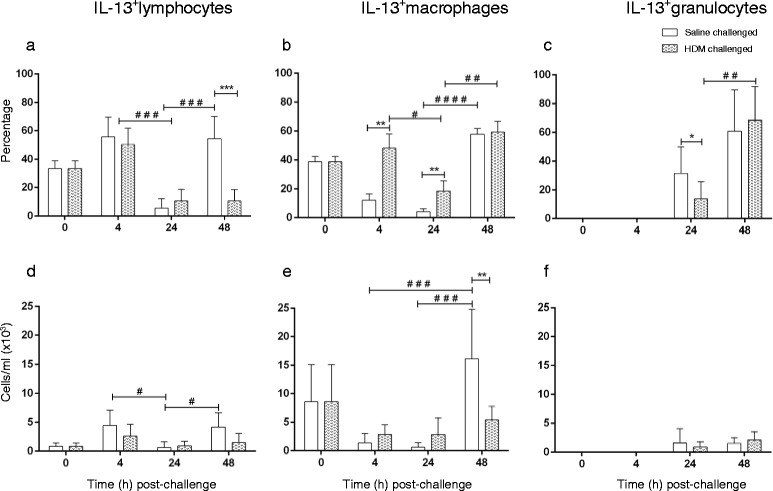
IL-13 detection in BAL cells following saline and HDM bronchial challenges. **a**–**c** percentage and (**d**–**f**) total numbers (cells/ml BAL) of IL-13 expressing lymphocytes, macrophages and granulocytes collected at 0 h (baseline), and at 4, 24 and 48 h post- segmental airway challenge. Data presented as mean positive cells (within subpopulation) ± SD for *n* = 6 sheep (*denotes significant differences; **p < 0.01, ***p < 0.001; ^#^denotes significant difference between time points: ^#^ *p* < 0.05, ^##^ *p* < 0.01, ^###^ *p* < 0.001, ^####^ *p* < 0.0001)

A significant decline in the proportion of lymphocytes expressing IL-13 was seen in both saline- and HDM- challenged lobes from 4 to 24 h post challenge followed by an increase in saline-challenged lungs at 48 h (Fig. 6a); a similar pattern of change in cell number was seen following saline challenge (Fig. 6d). The percentage of IL-13^+^ macrophages in HDM-challenged lobes was higher than in saline-challenged lobes at 4 and 24 h post-challenge (Fig. 6b). In HDM-challenged lungs, there was a decrease in the proportion of macrophages positive for IL-13 from 4 to 24 h, followed by an increase in the proportion (but not total cell number) of both macrophages and granulocytes at 48 h (Fig. 6b, c, e & f). The total number of IL-13 expressing macrophages was not significantly altered in HDM-challenged lobes but the number of these cells in saline-challenged lobes increased at 48 h compared to 4 and 24 h (Fig. 6e).

### IL-4 and IL-13 expression in lung tissue

**Fig. 7:**
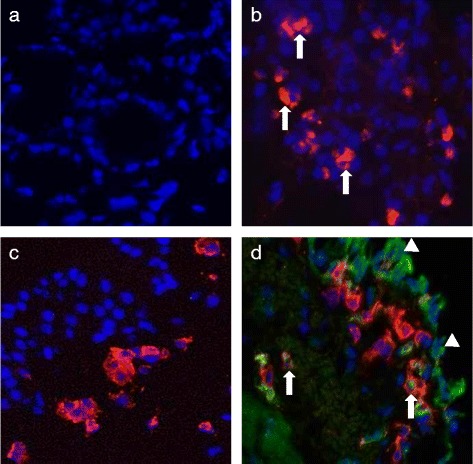
Immunostaining of IL-4 and IL-13 in lung tissues following allergen challenge, showing (**a**) negative staining (isotype-matched control Ab), (**b**) IL-4^+^ cells (red, arrows), (**c**) CD45^+^ leukocyte (red) staining, and (d) CD45^+^ (red) and IL-13^+^ (green, *arrows*) staining; the arrow shows an IL-13 expressing leukocyte (IL-13^+^CD45^+^) and the arrow head shows an IL-13 expressing non-leukocyte (IL-13^+^CD45^−^). Representative sections were taken from the same lung lobe of a sheep, collected post-mortem at 48 h post-HDM challenge. All slides were counterstained with DAPI (blue) when mounted in Mowiol (Original magnification × 400)

Post-mortem lung tissues were collected 48 h after a HDM allergen challenge and frozen tissue sections cut for immunostaining to examine IL-4 and IL-13 expression (Fig. 7). Intracellular expression of IL-4 and IL-13 was observed in cells within the lung tissues, and this was localized particularly within and below the epithelial layer (Fig. 7b and d). Dual staining of the lung tissue sections for IL-13 and CD45 (leukocyte common antigen) clearly showed that IL-13 was expressed in both immune (IL-13^+^CD45^+^) and non-immune (IL-13^+^CD45^−^) cells within the HDM-challenged airway tissues (Fig. 7d).

## Discussion

The pathophysiological features of allergic asthma are characterized by airway inflammation and structural and functional changes in the lung. These responses are directed by the actions of a number of key cytokines. IL-4 and IL-13 are two such cytokines that have been shown to play a central role in directing the pathophysiological changes in allergic asthma [3]. The role of Th_2_ cytokines has been extensively studied in small animal models of asthmatic disease and much of our current understanding arises from basic mechanistic, knockout and transgenic studies, and therapeutic interventions [26]. There is clear potential and much interest in the development of targeted anti-Th_2_ cytokine therapies for asthma [33]. However, mixed success with regard to anti-IL-4 and anti-IL-13 interventions in clinical trials to date [19, 20] confirms the need for a better understanding of the mechanisms of pathogenesis underlying the different Th2 ‘endotypes’ seen in asthma [34–37]. The ability to perform detailed kinetic studies and real-time measurements as well as dose—response efficacy studies and therapeutic interventions, requires the development and validation of large animal models that provide clarity of disease onset and progression. Such investigations will help to improve our understanding of the role for IL-4 and IL-13 in human asthmatic disease and identify effective, targeted therapeutic strategies [22].

There is comparatively little known about the role for Th_2_ cytokines in allergic airways disease in larger animal models such as dog, sheep, horse and non-human primates, although it appears Type 2 or Th_2_ immune pathways are involved, establishing some parallels between these larger animal models and human allergic asthma [26, 38–40]. In the present study we used a sheep model of allergic asthma to investigate the kinetics of expression of IL-4 and IL-13 in allergen-challenged airways, with the goal to extend our understanding of Th_2_-driven mechanisms in this large animal model system.

IL-4 and IL-13 are required for the IgE class switching in B lymphocytes [41], and increased BAL levels of IL-4 and IL-13 are consistent with elevated allergen-specific IgE in serum [23, 28] and BAL fluid [28] in the sheep asthma model. The early elevation of these Th_2_ cytokines followed by the recruitment of neutrophils, lymphocytes and macrophages in this model, precedes the late phase allergic response associated with the appearance of eosinophils from 24 to 48 h after allergen exposure. The fact that eosinophils were detected in saline-treated control lung lobes might suggest some degree of a systemic (ie lung-wide) response to segmental lung allergen exposure in this model. Indeed, altered BAL cellularity in control lobes has also been observed by others that have used repeated segmental airway challenge to study inflammation/infection in sheep [42]. In our own experience and that of others [43], it has been shown in sheep that repeated BAL sampling has no effect itself on BAL cellularity over time, and sampling from different lung lobes is comparable.

We found elevated IL-4 levels in BAL across all time-points examined following allergen challenge. Lymphocytes, most likely CD4^+^ T cells [23], were a major source of IL-4 in the immediate hours (4 h) after allergen challenge, while IL-4 expressing alveolar macrophages (AMs) were predominant in BAL at the later (24 and 48 h) time-points. IL-13 was elevated in BAL at 4 h post challenge, with lymphocytes (4 h) and AMs (4–48 h) found to be the major sources of IL-13 post allergen challenge. The immunostaining of airway tissue showed that the bronchial epithelium also serves as a cellular source for IL-13 in sheep airway tissues following allergen challenge, similar to that reported elsewhere [44]. Eosinophils and mast cells may also be a source of IL-4 and IL-13 [3, 4], and in this study and earlier investigations these cells have been shown to be a key feature of the inflammation seen in the sheep model of allergic asthma [22]. Indeed, the greater levels of intracellular IL-13 expression in BAL granulocytes from 24 to 48 h post-allergen challenge is associated with an increased eosinophil recruitment into BAL at this time. While not shown in the present study, a significant correlation between IL-13 expression and eosinophils in BAL has been reported elsewhere [14, 15].

The elevated IL-4 and IL-13 levels seen in the present study reinforces the idea that Th_2_ lymphocytes are key cellular players in directing the early immune response to allergen re-exposure in the sheep asthma model. Moreover, our observations with respect to the cellular source of these cytokines also implicate significant changes in the airway macrophage population and their involvement in the pathophysiology of allergic airway responses in sheep.

AMs are the principal cellular sentinels of the respiratory tract that are continually exposed to potentially inflammatory stimuli, and there is good evidence to suggest they become differentially activated during asthma responses [45]. Both IL-4 and IL-13 are involved in the alternative activation, or differentiation of the ‘M2 class’, of macrophages [46, 47] and recent studies have identified important roles for airway macrophages in allergic inflammation, including promotion of Th_2_ reactivity and airway tissue remodelling [48, 49]. M2 macrophages have been shown to be more abundant in the BAL and airway tissues of asthmatics compared with healthy subjects [50]. Further, enhanced levels of IL-13 producing macrophages have been found in the BAL from subjects with severe asthma, suggesting that M2 macrophages may contribute to reduced lung function in asthma patients [51].

In the present study, there was an initial decline observed in AM numbers following allergen challenge but also a clear shift in their functional phenotype, with an increase in the proportion of IL-4 (24 h) and IL-13 (4 and 24 h) producing AMs, suggesting the emergence of alternatively activated macrophages [52, 53] and a likely contribution to the key pathophysiological features of the sheep allergic asthma model. We have also found in preliminary studies that AMs collected from sheep asthmatic airways are altered in their ability to release cytokines in response to various stimuli, and display a diminished capacity for phagocytosis *ex vivo* (Liravi et al., unpublished data), typical of the changes seen in alveolar macrophages in asthma [54]. While it was shown that IL-13 expression in AMs was elevated at 4 and 24 h in allergen challenged airways, we found a similar proportion of IL-13 expressing AMs in saline and allergen-challenged airways at 48 h. Constitutive expression of IL-13 by AMs in normal subjects and elevated expression in subjects with airway disease may reflect the broad role for IL-13 in homeostasis and disease [49, 55].

IL-4 is known to direct the differentiation of AMs and their release of pro-inflammatory cytokines such as IL-6 and TNFα [56]. In the present study TNFα levels in BAL showed minimal change following allergen or saline exposure, however BAL IL-6 levels were elevated at all time-points in response to allergen, with Th_2_ lymphocytes, AMs and/or airway epithelial cells [31] the most likely source. Indeed, the inflammatory pathways underlying mucus hyperplasia and AHR seen in the sheep asthma model may be partly dependent on IL-6 signalling, although the contribution of classical or IL-6 trans-signalling, as recently investigated in mice and human subjects [57], is yet to be elucidated in the sheep model. IL-10 levels in BAL were highest at 4 h but lower at 48 h following allergen challenge compared to saline challenge. In the present study we did not investigate the cellular source of IL-10. However, sheep AMs are able to produce IL-10 in response to IL-13 *ex vivo* (Liravi et al., unpublished data) and macrophages from asthmatics are known to secrete elevated IL-10 levels [58]. On the other hand, Tregs (regulatory T lymphocytes) could also be a source of IL-10, although these cells are less functional and their numbers are reduced in asthma [59].

IL-4 and IL-13 overwhelmingly have an impact on airway inflammation, directing the recruitment and activation of immune cells including airway dendritic cells, alveolar macrophages, eosinophils, mast cells and neutrophils, mucin production by airway epithelial cells and airway wall remodelling in response to allergen exposure [4, 12]. These features have all previously been reported in the sheep asthma model [24, 28], and together with findings from our present study on cytokine data not previously documented for any other large animal model of asthma, strongly implicates a role for Th_2_ cytokines in the inflammatory and disease processes in this model system.

Future studies in the sheep asthma model may investigate the changes in expression and subsequent therapeutic targeting of other Th_2_-associated mediators such as IL-17, IL-22, IL-25 and IL-33 [60–63], or miRNAs thought to play a role in directing the pathways of IL-4 and/or IL-13 production in allergic airways, including miR-21, miR-145 and miR-155 [64–67].

## Conclusion

The results of this study provide further insight into the kinetics of cytokine expression in allergen-challenged airways, and for the first time in a large animal model demonstrate a Th_2_ polarized cytokine profile featuring IL-4 and IL-13 associated with allergen-induced airway inflammation.
